# Structure-Function Relationships in PMA and PMAT Series Copolymers for Polymer Solar Cells

**DOI:** 10.3390/polym10040384

**Published:** 2018-04-01

**Authors:** Jhe-Han Chen, Chi-Kan Liu, Wei-Che Chang, Pai-Tao Sah, Li-Hsin Chan

**Affiliations:** Department of Applied Materials and Optoelectronic Engineering, National Chi Nan University, Nantou 54561, Taiwan; s102328505@mail1.ncnu.edu.tw (J.-H.C.); s101328511@mail1.ncnu.edu.tw (C.-K.L.); s102328025@mail1.ncnu.edu.tw (W.-C.C.); s102328040@mail1.ncnu.edu.tw (P.-T.S.)

**Keywords:** two-dimensional donor-acceptor copolymer, 3,4-bis(4-bromophenyl)maleimide, side chain triphenylamine, acceptor end groups

## Abstract

Two series (PMA and PMAT) of two-dimensional donor-acceptor copolymers consisting of a 3,4-bis(4-bromophenyl)maleimide derivative and triphenylamine with a conjugated side chain were designed and synthesized to probe their structure-function relationships for use in bulk heterojunction (BHJ) polymer solar cells (PSCs). The difference between PMA- and PMAT-series is the conjugated side chain length on the triphenylamine unit. By extending the side chain length, and by attaching various acceptor end groups to the side chain, the electronic and photophysical properties of these copolymers, as well as subsequent device performance, were significantly affected. Two series of copolymers showed broad absorption in the visible region with two obvious peaks. With increasing electron-withdrawing strength of the acceptor end groups, the intramolecular charge transfer peak becomes progressively red-shifted. Highest occupied molecular orbital (HOMO) levels in each copolymer series are similar, but lowest unoccupied molecular orbital (LUMO) levels are dictated by the acceptors. BHJ PSCs composed of the copolymers as a donor and [6,6]-phenyl-C71-butyric acid methyl ester (PC_71_BM) as an acceptor in 1:2 weight ratio were fabricated and characterized. PSCs based on PMA- and PMAT-series copolymers had power conversion efficiencies (PCEs) ranging from 2.05–2.16% and 3.14–4.01%, respectively. These results indicate that subtle tuning of the chemical structure can significantly influence PSC device performance.

## 1. Introduction

Significant research attention has been focused recently on polymer solar cells (PSCs) based on a blended system of an electron donor polymer and an electron acceptor of fullerene derivative (for example, [6,6]-phenyl C61 butyric acid methyl ester (PC_61_BM) or [6,6]-phenyl C71 butyric acid methyl ester (PC_71_BM)) with nanoscale phase-separated bulk heterojunction (BHJ) morphology, due to their low cost, light weight, and flexibility for large-area devices [1,2,3]. Significant advances in power conversion efficiencies (PCEs) have been achieved in PSCs; more than 10% for traditional single-junction PSCs and over 11% for tandem PSCs [4,5,6,7]. A vast amount of research has attempted to implement a BHJ PSC with high efficiency, in which the conjugated polymer donor material is thought to play an important role in determining overall device performance. 

Several criteria for designing a conjugated polymer with high PCE have been proposed, including: (i) reducing the bandgap of the polymer to broaden the absorption region to sunlight, which is beneficial for obtaining a high short-circuit current (*J*_sc_) [8,9]; (ii) modulating the lowest unoccupied molecular orbital (LUMO) energy level of the polymer to appropriately match the acceptor [8]; (iii) lowering the highest occupied molecular orbital (HOMO) level of the polymer to facilitate high open-circuit voltage (*V*_oc_) [9]; (iv) achieving a nanoscale interpenetrating network morphology for efficient charge separation and transport [10,11]; and (v) adding electron-donating and/or electron-accepting moieties into the polymer structure to enhance the charge carrier mobilities for increased charge collection and reduced charge recombination [12]. Among the multiple strategies for designing conjugated polymers, many have attempted to combine the electron-rich and electron-deficient units into the polymer backbone to form donor-acceptor (D-A) systems, in which their HOMO and LUMO energy levels can be adjusted and the overall bandgap of polymer can be reduced [13,14,15,16]. Moreover, two-dimensional (2D) conjugated polymers, which possess conjugated side groups or side chains that may provide broadened absorption spectra, high hole mobility, and low-lying HOMO energy levels, have significant potential as conjugated donor materials in high-performance PSCs. For example, Li and coworkers first developed the concept of 2D conjugated polythiophenes; they found the 2D structure not only can broaden the absorption spectra but also enhance the hole mobility [17]. Cao and Huang reported that the bandgap and energy levels of silafluorene-based 2D conjugated polymers were effectively tuned through changing the electron-withdrawing strength of the acceptor groups on the side chains [18]. Hou’s group reported a series of 2D-conjugated polymers based on benzo[1,2-*b*:4,5-*b*’]dithiophene (BDT) and thienothiophene units, in which the side groups of BDT were varied using furan, thiophene or selenophene, the photovoltaic performance of the resultant polymers were significantly affected [19]. Shen et al. reported by introduction of strong electron-withdrawing groups, such as pyrrolo[3,4-*c*]pyrrole-1,4-dione into the 2D-conjugated polymer structures, leading to broaden the spectral absorption of the polymers [20]. Recently, Cho and coworkers reported high PCEs of devices were achieved by extending the π-conjugation side chains with oligothienyl groups on 2D D-A copolymers [21].

On the basis of the above considerations, two series (PMA and PMAT) of two-dimensional donor-acceptor conjugated copolymers, consisting of a 3,4-bis(4-bromophenyl)maleimide derivative and triphenylamine with a conjugated side chain with various acceptors attached as end groups, are designed and synthesized to probe their structure-function relationships for use in BHJ PSCs. The electron-deficient maleimide derivative is one of the larger types of heterocyclic aromatic compound (like phthalimide, naphthalimide, and peryleneimide), which have been demonstrated as the building blocks for constructing organic transistors, organic light-emitting diodes, and PSCs [22,23,24,25,26,27]. Triphenylamine has some beneficial features, such as convenient chemical modification, a propeller-like molecular structure, and strong electron-donating and hole-transporting capabilities, and is a promising building block for optoelectronic materials [28]. The selection of acceptors on the conjugated side chain of triphenylamine unit is based on the electron-withdrawing strength and commercial availability. Therefore, we employed the maleimide derivative as an electron-withdrawing unit while triphenylamine with a side chain was used as an electron-donating unit when constructing the copolymers. The resulting copolymers exhibit 2D D‒A structures with broad absorption region. In this study, the influence of various side chain lengths and acceptor end groups of the triarylamine moiety on the copolymers’ photophysical, electrochemical, and photovoltaic properties are investigated in detail. The morphological and photovoltaic characteristics of the copolymer/fullerene derivative-blend films are also discussed. This research demonstrates that various side chain lengths and end groups in the copolymer structure significantly influence the absorption range, electronic energy levels, and morphologies of thin films and potentially act as active layer materials for PSCs applications.

## 2. Experimental Details

### 2.1. Materials

Unless noted, all solvents and chemicals were purchased from Aldrich (Saint Louis, MO, USA), Alfa Aesar (Haverhill, MA, USA), and TCI Chemical Co., (Tokyo, Japan) and were used as received. Diethyl ether, tetrahydrofuran (THF), *N*,*N*-dimethylmethanamide (DMF), and toluene were dried using drying agents and freshly distilled before use. *N*-(2-ethylhexyl)-3,4-bis{4-[(3-octylthien-2-yl)phenyl]}maleimide (TML) was synthesized using methods in our previous reports [27,29,30,31]. 4-[*N*,*N*-di(4-bromophenyl)amino]benzaldehyde (TA) and 2-(2-{4-[*N*,*N*-di(4-bromophenyl)amino]phenyl}ethenyl)thien-5-al (TAT) were prepared according to procedures reported in the literature [32,33,34]. [6,6]-Phenyl C61 butyric acid methyl ester (PC_61_BM), [6,6]-phenyl C71 butyric acid methyl ester (PC_71_BM), and poly(3,4-ethylenedioxythiophene):poly(styrenesulfonate) (PEDOT:PSS, Clevios P-VP AI4083) were purchased from Uni-onward corporation and used as received.

#### 2.1.1. Synthesis of PMA-CHO

The polymerization procedure was performed according to methods described in the literature [35]. A mixture of *N*-(2-ethylhexyl)-3,4-bis{4-[(3-octylthien-2-yl)phenyl]}maleimide (TML) (490 mg, 0.65 mmol), 4-[*N*,*N*-di(4-bromophenyl)amino]benzaldehyde (TA) (280 mg, 0.65 mmol), Pd(OAc)_2_ (7.3 mg, 5 mmol%), K_2_CO_3_ (230 mg, 1.66 mmol), PCy_3_·HBF_4_ (24.7 mg, 10 mmol%), and PivOH (67 mg, 0.65 mmol) were dissolved in a solution of anhydrous *N*,*N*-dimethylacetamide (DMAC) (2.5 mL) and xylene (5 mL) in a two-necked flask under a nitrogen atmosphere. The mixture was heated to 110 °C with vigorous stirring and the reactions proceeded for 24 h. After cooling down, the mixture was poured into a solution (120 mL) of methanol and deionized water (5:1). The precipitate was collected by filtration through a funnel and then dissolved in chloroform, and reprecipitated in methanol. Further purification was conducted by Soxhlet-extracted method using *n*-hexane and methanol. The resulting polymer PMA-CHO was obtained as a dark red solid with isolated yields of 65% after drying in a vacuum for 24 h. ^1^H NMR (300 MHz, CD_2_Cl_2_, δ/ppm): 9.81 (s, 1H), 7.81–6.92 (m, 22H), 3.56 (d, 2H, *J* = 6.0 Hz), 2.70 (d, 4H, *J* = 6.0 Hz), 1.81(br, 1H), 1.66 (br, 4H), 1.34–1.24 (m, 28 H), 0.96–0.83 (m, 12 H). ^13^C NMR (75 MHz, CDCl_3_, δ/ppm): 190.74, 171.41, 152.96, 145.53, 142.26, 139.80, 136.71, 136.53, 135.34, 133.16, 131.66, 131.22, 130.50, 130.15, 129.20, 129.25, 127.90, 127.77, 127.09, 126.45, 124.65, 120.78, 42.60, 38.74, 32.14, 31.22, 30.89, 29.83, 29.70, 29.43, 29.14, 28.92, 24.23, 23.29, 22.95, 14.40, 10.76.

#### 2.1.2. Synthesis of PMAT-CHO

PMAT-CHO was synthesized with the same method used for PMA-CHO, using TML (563 mg, 0.75 mmol), 2-[2-[4-[*N*,*N*-di(4-bromophenyl)amino]phenyl] ethenyl]thien-5-al (TAT) (405 mg, 0.75 mmol), Pd(OAc)_2_ (8.4 mg, 5 mol %), K_2_CO_3_ (264 mg, 1.91 mmol), PCy_3_·HBF_4_ (28.5 mg, 10 mol %), and PivOH (77 mg, 0.75 mmol) in a solution of anhydrous DMAC (3 mL) and xylene (6 mL). The resulting polymer was a dark red solid with a yield of 75% after drying in a vacuum for 24 h. ^1^H NMR (300 MHz, CD_2_Cl_2_, δ/ppm): 9.83 (d, 1H, *J* = 9.3 Hz), 7.70–6.75 (m, 26H), 3.53 (d, 2H, *J* = 3.6 Hz), 2.68 (br, 4H), 1.80 (br, 1H), 1.60 (br, 4H), 1.45–1.04 (m, 28H), 0.84–0.94 (m, 12H). ^13^C NMR (75 MHz CDCl_3_, δ/ppm): 182.77, 171.41, 146.50, 142.63, 141.42, 140.98, 139.78, 137.12, 136.63, 136.26, 135.37, 130.46, 130.17, 129.51, 129.20, 128.90, 127.78, 126.85, 125.87, 125.11, 124.64, 42.57, 38.72, 32.13, 31.23, 30.88, 29.82, 29.68, 29.52, 28.91, 24.23, 23.28, 22.94, 14.40, 10.76.

#### 2.1.3. Synthesis of PMA-DCN

A mixture of PMA-CHO (60 mg) and malononitrile (120 mg, 1.82 mmol) was dissolved in a solution of 0.4 mL of pyridine and 8 mL of chloroform. The mixture was stirred at 40 °C and the reactions proceeded for 24 h. After cooling down, the mixture was poured into *n*-hexane and the precipitate was collected by filtration through a funnel. Further purification was conducted by Soxhlet extractions using *n*-hexane. The resulting polymer PMA-DCN was obtained as a dark red solid (16 mg) after drying in a vacuum. ^1^H NMR (300 MHz, CD_2_Cl_2_, δ/ppm): 7.85–6.91 (m, 23H), 3.56 (d, 2H, *J* = 5.7 Hz), 2.71 (d, 4H, *J* = 6.3 Hz), 1.81 (br, 1H), 1.66 (br, 4H), 1.45–1.10 (m, 28H), 0.94–0.83 (m, 12H). ^13^C NMR (75 MHz CDCl_3_, δ/ppm): 171.40, 158.10, 153.04,145.25, 144.50, 141.94, 141.11, 139.82, 137.09, 136.87, 136.46, 135.36, 133.28, 132.21, 130.52, 130.40, 130.20, 129.53, 129.27, 128.22, 127.98, 127.75, 127.23, 126.95, 126.58, 126.07, 124.99, 124.67, 123.82, 119.85, 119.31, 115.34, 114.24, 42.60, 38.74, 32.14, 31.22, 30.89, 29.83, 29.70, 29.53, 29.42, 29.15, 28.92, 24.24, 23.29, 22.95, 14.41, 10.77.

#### 2.1.4. Synthesis of PMA-CNR

The synthetic procedure of PMA-CNR was similar to that for PMA-DCN, using PMA-CHO (60 mg) and octyl cyanoacetate (120 mg, 0.60 mmol) in a solution of 0.4 mL of pyridine and 8 mL of chloroform. The mixture solution was stirred at 40 °C for 24 h. Then, the resulting mixture was poured into acetone and the precipitate was filtered off. The precipitate was collected and Soxhlet-extracted with acetone and dried in a vacuum, generating PMA-CNR (28 mg) as a very dark red solid. ^1^H NMR (300 MHz, CD_2_Cl_2_, δ/ppm): 8.09 (br, 1H), 7.87 (br, 2H), 7.80–6.85 (m, 20H), 4.25 (s, 2H), 3.55 (d, 2H, *J* = 4.5 Hz), 2.71 (br, 4H), 1.80 (br, 1H), 1.66 (br, 4H), 1.50–1.10 (m, 42 H), 0.94–0.83 (m, 15H). ^13^C NMR (75 MHz CDCl_3_, δ/ppm): 171.42, 163.85, 154.05, 151.98, 145.09, 142.18, 141.05, 139.80, 137.11, 136.83, 136.53, 135.37, 133.44, 131.56, 130.49, 130.18, 129.52, 129.26,127.89, 127.12, 126.60, 126.39, 124.66, 120.57, 116.86, 98.37, 66.75, 42.59, 38.73, 32.14, 32.07, 31.22, 30.88, 29.83, 29.70, 29.52, 29.45, 29.15, 28.91, 28.85, 26.10, 24.22, 23.30, 22.95, 14.41, 10.76.

#### 2.1.5. Synthesis of PMA-CNB

A mixture of PMA-CHO (150 mg), benzothiazole-2-acetonitrile (300 mg, 1.72 mmol), and Al_2_O_3_ (150 mg, 1.45 mmol) was dissolved in 5 mL of toluene. The solution was heated to 110 °C with vigorous stirring and the reactions proceeded for 24 h. Then, the resulting mixture was poured into acetone and the precipitate was filtered off. The precipitate was collected by filtration, Soxhlet-extracted with acetone, and dried in a vacuum, generating PMA-CNB (60 mg) as a dark red solid. ^1^H NMR (300 MHz, CD_2_Cl_2_, δ/ppm): 8.01–7.80 (m, 5H), 7.70–6.92 (m, 22H), 3.54 (s, 2H), 2.69 (s, 4 H), 1.80 (br, 1H), 1.65 (br, 4H), 1.40–1.13 (m, 28H), 0.93–0.83 (m, 12H). ^13^C NMR (75 MHz CDCl_3_, δ/ppm): 171.43, 163.99, 153.99, 151.08, 146.20, 145.35, 142.28, 141.06, 139.80, 136.72, 136.56, 135.34, 135.10v132.53, 131.27, 130.88, 130.49, 130.19, 129.52, 129.26, 127.87, 127.09, 126.65, 126.41, 125.86, 125.65, 125.21, 124.66, 123.48, 121.89, 121.15, 117.70, 101.66, 42.59, 38.74, 32.15, 31.24, 30.88, 29.85, 29.71, 29.54, 29.15, 28.82, 24.23, 23.30, 22.96, 10.77.

#### 2.1.6. Synthesis of PMAT-DCN

PMAT-DCN was prepared using the same procedures as described for PMA-DCN using PMAT-CHO (60 mg) and malononitrile (120 mg, 1.82 mmol) in a solution of 0.4 mL of pyridine and 8 mL of chloroform. The mixture was stirred at 40 °C and the reactions proceeded for 24 h. After cooling to room temperature, the mixture was poured into n-hexane and the precipitate was collected by filtration through a funnel. Following the same procedure, Soxhlet extractions was conducted using *n*-hexane, and PMAT-DCN (36 mg) was obtained as a very dark red solid after drying in a vacuum. ^1^H NMR (300 MHz, CD_2_Cl_2_, δ/ppm): 7.80–6.75 (m, 27H), 3.54 (s, 2H), 2.68 (s, 4H), 1.79 (br, 1H), 1.65 (br, 4H), 1.41–1.02 (m, 28H), 0.91–0.84 (m, 12H). ^13^C NMR (75 MHz CDCl_3_, δ/ppm): 171.42, 150.38, 148.58, 146.28, 142.54, 141.000, 140.57, 139.57, 137.11, 136.82, 136.57, 136.36, 135.37, 134.73, 133.52, 131.54, 130.47, 130.18, 129.51, 129.20, 128.90, 128.69, 127.78, 126.90, 125.96, 125.40, 124.64, 123.37, 118.59, 114.78, 113.97, 42.57, 38.73, 32.13, 31.23, 30.88, 29.82, 29.68, 29.52, 29.14, 28.90, 24.23, 23.28, 22.94, 10.76.

#### 2.1.7. Synthesis of PMAT-CNR

PMAT-CNR was prepared using the same procedures as described for PMA-CNR using PMAT-CHO (60 mg) and octyl cyanoacetate (120 mg, 0.60 mmol) in a solution of 0.4 mL of pyridine and 8 mL of chloroform. The mixture solution was stirred at 40 °C for 24 h. Then, the resulting mixture was poured into acetone and the precipitate was filtered off. The precipitate was collected and Soxhlet-extracted with acetone and dried in a vacuum to generate PMAT-CNR (34 mg) as a blackish red solid. ^1^H NMR (300 MHz, CD_2_Cl_2_, δ/ppm): 8.23 (br, 1H), 7.77–6.81(m, 26H), 4.24 (s, 2H), 3.53 (s, 2H), 2.68 (s, 4H), 1.80–1.45 (br, 5H), 1.40–1.05 (br, 42H), 0.90–0.83 (m, 15H). ^13^C NMR (75 MHz CDCl_3_, δ/ppm): 171.41, 163.41, 148.15, 146.44, 142.64, 140.97, 139.77, 137.10, 136.63, 136.27, 135.36, 134.33, 133.19, 130.46, 130.17, 129.50, 129.19, 128.89, 128.44, 127.78, 126.87, 125.90, 125.20, 124.63, 119.43, 116.49, 110.25, 97.48, 66.81, 42.56, 38.72, 32.12, 32.05, 31.22, 30.88, 29.81, 29.68, 29.51, 29.44, 29.13, 28.89, 26.08, 24.23, 23.28, 10.75.

#### 2.1.8. Synthesis of PMAT-CNB

Following the same procedures for PMA-CNB synthesis, PMAT-CNB was obtained using PMAT-CHO (150 mg), benzothiazole-2-acetonitrile (300 mg, 1.72 mmol), and Al_2_O_3_ (150 mg, 1.45 mmol) in 5 mL of toluene. The mixture solution was heated to 110 °C with vigorous stirring and the reactions proceeded for 24 h. Then, the resulting mixture was poured into acetone and the precipitate was filtered off. The precipitate was collected by filtration and Soxhlet-extracted with acetone and dried in a vacuum, generating PMAT-CNB (85 mg) as a very dark red solid. ^1^H NMR (300 MHz, CD_2_Cl_2_, δ/ppm): 8.15–6.65 (m, 31H), 3.53 (s, 2H), 2.66 (s, 4H), 1.80 (br, 1H), 1.45–1.01 (m, 28H), 0.91–0.82 (m, 12H). ^13^C NMR (75 MHz CDCl_3_, δ/ppm): 171.42, 163.05, 154.04, 147.55, 146.49, 142.82, 141.00, 139.79, 138.02, 137.65, 137.12, 136.64, 135.23, 132.50, 131.34, 130.47, 130.18, 129.82, 129.51, 129.20, 128.90, 128.35, 127.79, 127.09, 125.90, 124.65, 123.90, 123.47, 121.92, 119.63, 117.39, 110.26, 42.57, 38.74, 32.14, 31.24, 30.89, 29.83, 29.69, 29.53, 29.15, 28.92, 24.24, 23.29, 22.95, 10.76.

### 2.2. Characterization of Copolymers

^1^H NMR and ^13^C NMR spectra were collected on a Varian Unity Inova 300 WB NMR workstation. Gel-permeation chromatography was performed on a Waters GPC-1515 using THF as the eluent and polystyrene as a reference sample to determine the molecular weights of copolymers. Thermogravimetric analyses were performed on a Perkin-Elmer TGA-7 analyzer under nitrogen atmosphere at a heating rate of 10°/min from 30 to 800 °C, and the thermal decomposition temperatures (*T*_d_) of the copolymers were determined when a 5% weight loss occurred. UV-visible absorption spectra were recorded on a Hewlett-Packard 8453 spectrophotometer. Cyclic voltammetry (CV) was conducted on an electro-chemical analyzer CHI 612D at a scanning rate of 50 mV s^−1^ in an anhydrous N_2_-saturated solution of 0.1 M Bu_4_NClO_4_ in MeCN. A Pt plate coated with a thin polymer film was used as the working electrode, a Pt wire was the counter electrode, and an Ag/Ag^+^ electrode was the reference electrode. The electrochemical potential was calibrated against Ferrecene/Ferrocene^+^. The surface morphology of the active layers was determined by transmission electron microscopy (TEM) using a JEOL JEM-1200EX II instrument at an accelerating voltage of 120 kV.

### 2.3. Fabrication and Characterization of PSCs

All PSCs were prepared according to the following fabrication procedure. The patterned indium tin oxide (ITO) glass substrates (with a sheet resistance of 8 Ω/sq) were sonicated and sequentially cleansed with detergent, deionized water, acetone, and isopropyl alcohol. Prior to device fabrication, the cleaned glass substrates were dried on a hot plate, and treated with oxygen plasma for 5 min. The hole-transporting material poly(3,4-ethylenedioxythiophene):poly(styrenesulfonate) (PEDOT:PSS, Clevios P-VP AI4083) was spin-coated on the ITO-coated glass at a spinning rate of 4000 rpm for 60 s. The sample was then dried on a hot plate at 120 °C for 10 min. Subsequently, the copolymer/PC_71_BM layer (with a weight ratio of 1:2) in *o*-dichlorobenzene (*o*-DCB) was formed on the top of the PEDOT:PSS layer by spin-coating (600 rpm, 60 s) the mixture solution and then annealed at 140 °C for 10 min. The cathode of Ca (30 nm)/Al (100 nm) film was deposited on the copolymer/PC_71_BM blend film by thermal evaporation in a high-vacuum chamber. The effective area of each device determined by a shadow mask was 4 mm^2^. The current density-voltage (*J*-*V*) characteristics of the PSCs were measured with a Keithley 2400 source meter under AM 1.5 G illumination at 100 mW cm^−2^ (Oriel solar simulator). The incident photon-to-electron conversion efficiency (IPCE) was measured using a monochromator connecting to a xenon lamp as the light source and a photodiode connecting to a lock-in amplifier to monitor its output. All electrical measurements were made in air. Hole-only devices were fabricated with the device configuration of ITO/PEDOT:PSS/polymer:PC_71_BM (1:2, *w*/*w*)/Au for determination of the hole mobilities of the blend films. The space-charge-limited-current (SCLC) hole mobility was calculated using the Mott-Gurney equation [36,37],

$$J=\frac{9}{8}\varepsilon \varepsilon_{0}\mu \frac{V^{2}}{L^{3}}$$

where *J* is the current density; *ε* is the relative permittivity of a blend film; *ε*_0_ is the permittivity of a vacuum; *V* is the applied voltage, and *L* is the thickness of the blend film.

## 3. Results and Discussion

### 3.1. Characterization of Copolymers

**Scheme 1:**
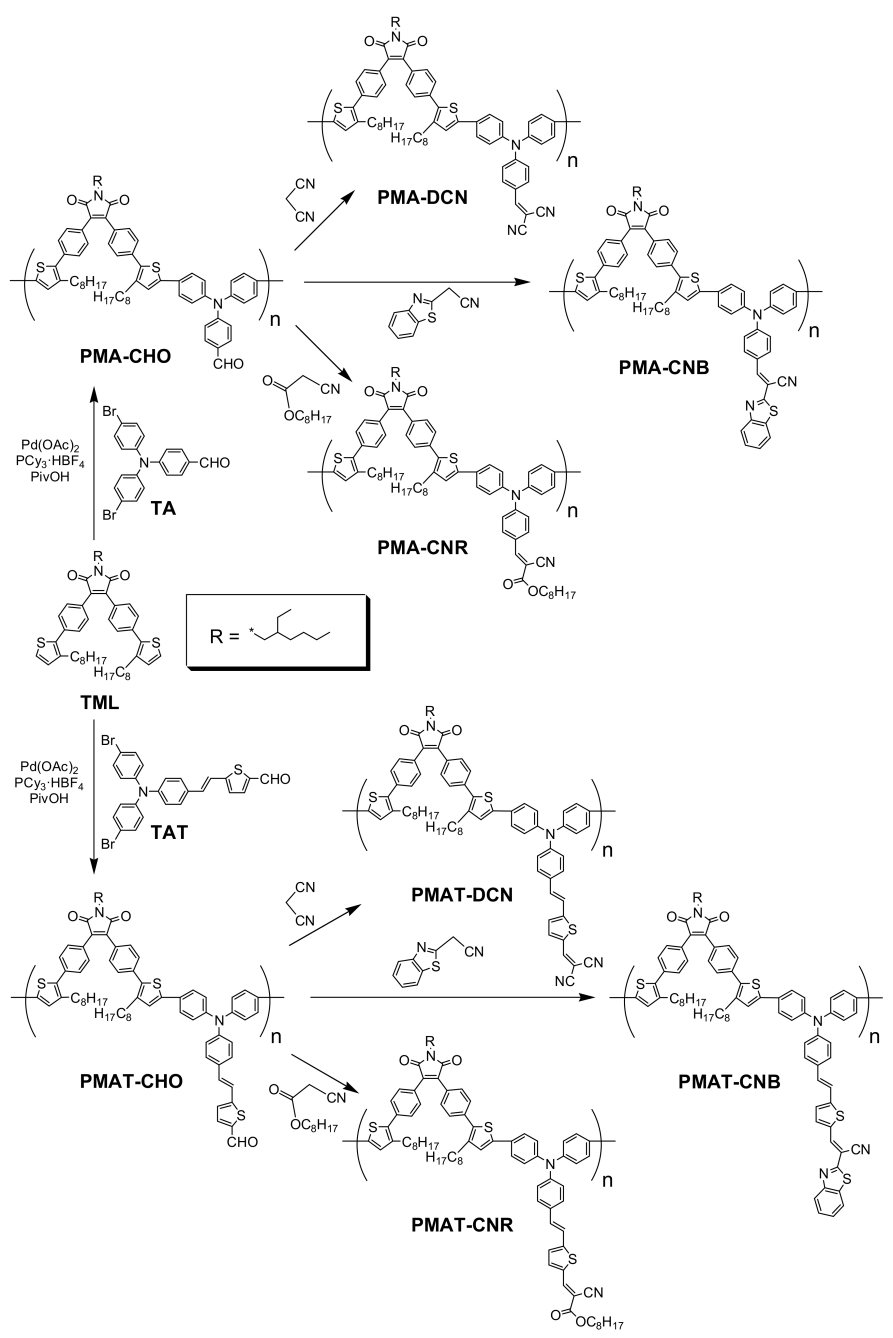
Synthetic scheme for PMA- and PMAT-series copolymers.

**Figure 1:**
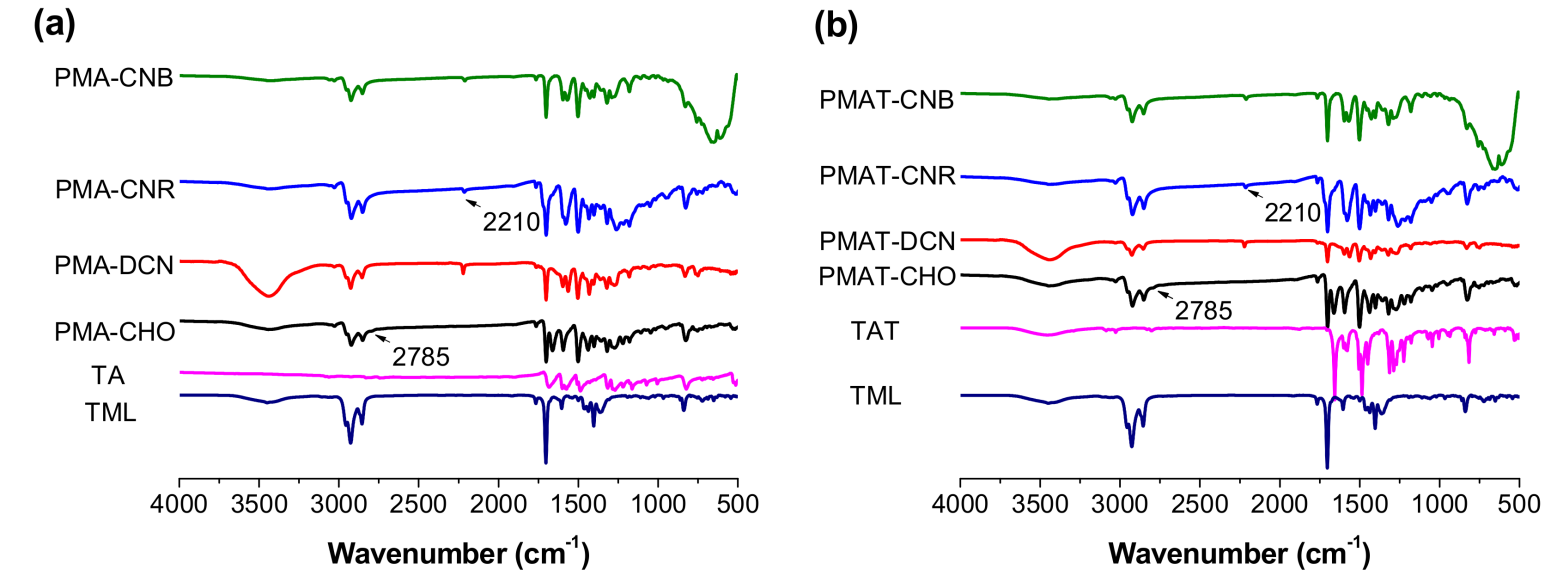
FT-IR spectra of (**a**) PMA-series and (**b**) PMAT-series. The monomers *N*-(2-ethylhexyl)-3,4-bis{4-[(3-octylthien-2-yl)phenyl]}maleimide (TML), 4-[*N*,*N*-di(4-bromophenyl)amino]benzaldehyde (TA) and 2-(2-{4-[*N*,*N*-di(4-bromophenyl)amino]phenyl}ethenyl)thien-5-al (TAT) are incorporated for comparison.

The synthetic routes of the PMA- and PMAT-series copolymers (including PMA-CHO, PMA-CNR, PMA-DCN, PMA-CNB, PMAT-CHO, PMAT-CNR, PMAT-DCN, and PMAT-CNB) are shown in Scheme 1. TML, TA, and TAT were synthesized according to procedures discussed in the literature [27,29,30,31,32,33,34]. Two precursor copolymers (PMA-CHO and PMAT-CHO) were obtained through Pd-catalyzed direct C–H arylation reaction consisting of TML and TA (or TAT) in equimolar ratio. The resulting copolymers, PMA-CHO and PMAT-CHO, were further functionalized through the Knoevenagel condensation reaction between aldehyde and various acceptor end groups to obtain PMA- and PMAT-series copolymers. The difference between PMA- and PMAT-series is the conjugated side chain length on the triphenylamine unit. To study the structure-function relationships, various acceptor end groups were designed for the PMA- and PMAT-series copolymers. To confirm conversion of the aromatic aldehyde functional group of PMA-CHO and PMAT-CHO to other acceptor end groups, Fourier transform infrared (FT-IR) absorption spectroscopy measurements were performed. Figure 1 depicts the FT-IR spectra of the copolymers; FT-IR spectra of the monomers, such as TML, TA and TAT, were also included for comparison. The characteristic absorption peaks of the aldehyde group that appeared at 1660 and 2785 cm^−1^, associated with C–H and C=O stretching vibrations, respectively, disappeared after converting to various acceptor end groups. In addition, a new absorption peak appeared at 2210 cm^−1^, corresponding to the C≡N stretching vibration, indicating that the cyano (–CN) acceptor end group was successfully attached on the copolymer. However, the absorption peak at approximately 1700 cm^−1^, corresponding to C=O stretching vibrations of the octyl cyanoacetate group for PMA-CNR and PMAT-CNR, overlapped with the C=O stretching of TML; therefore, C=O stretching vibrations of the octyl cyanoacetate group could not be identified in the FT-IR spectra [34]. The conversion of the aromatic aldehyde functional group of PMA-CHO and PMAT-CHO to octyl cyanoacetate end groups was also verified by ^1^H NMR. For example, the complete disappearance of the aromatic aldehyde proton signal at approximately 9.81 ppm confirmed the conversion reaction. In addition, appearance of the alkyl proton signal at 4.2 ppm indicated that the octyl cyanoacetate group was attached on the triphenylamine unit of the polymer.

The resulting copolymers were soluble in chlorinated solvents, such as chloroform, chlorobenzene, and *o*-DCB, which are important for application in PSCs. Table 1 presents the number-average (*M*_n_) and weight-average (*M*_w_) molecular weights of these polymers against polystyrene standards, as determined by GPC, with THF as the eluent. Various acceptor end groups attached on PMA-CHO or PMAT-CHO in the other three copolymers led to a slight increase in molecular weights and the corresponding polydispersity indices. The thermal stability of the conjugated polymer is essential for it is related to the operational stability of an optoelectronic device; therefore, a high *T*_d_ is preferred for application of a conjugated polymer to PSCs. As shown in Table 1, PMA- and PMAT-series copolymers had *T*_d_ values up to 418 and 421 °C, respectively, under a nitrogen atmosphere. These copolymers exhibited favorable thermal stability, which is sufficient for PSCs applications.

### 3.2. Optical Properties

**Figure 2:**
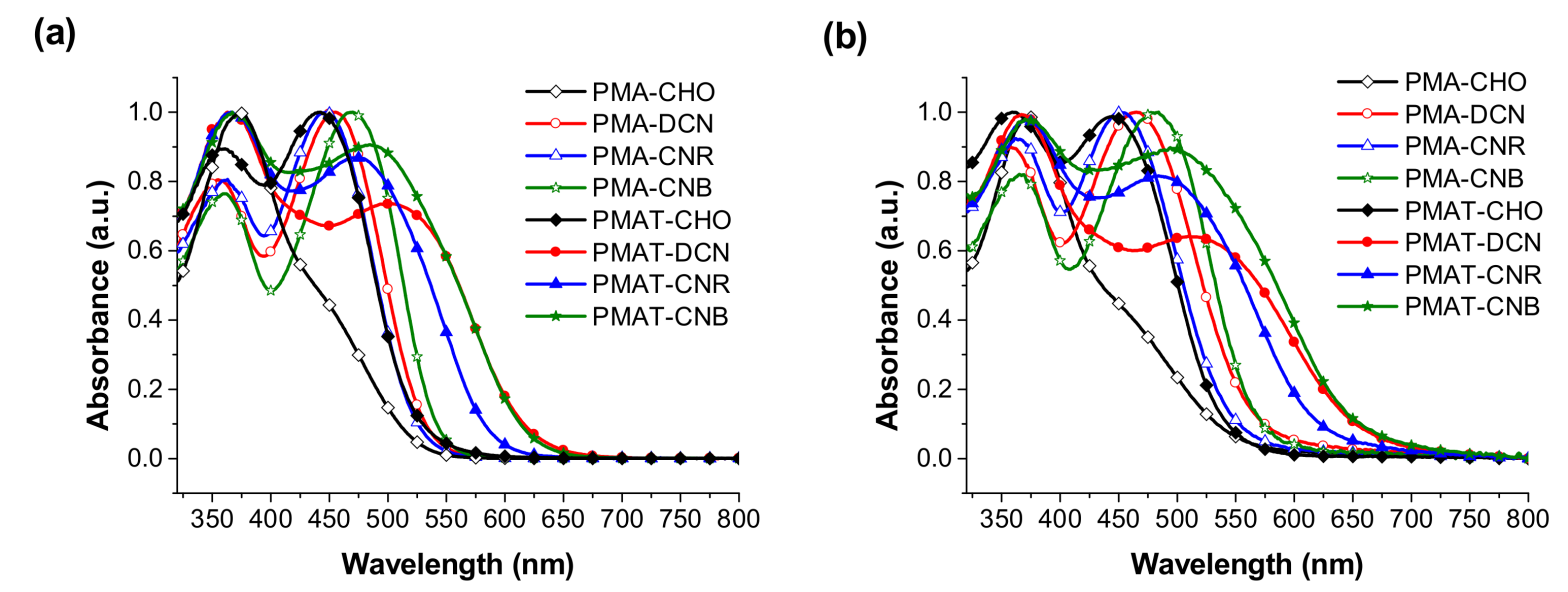
Ultraviolet (UV)-visible absorption spectra of the copolymers (**a**) in toluene solutions and (**b**) in thin films.

Figure 2 displays the absorption spectra of PMA- and PMAT-series copolymers in toluene solutions and thin films. Table 2 summarizes the corresponding absorption data of these copolymers. In solution, both copolymer series exhibited broad absorption and featured two clear absorption peaks, except for PMA-CHO. The first absorption band in the range of 330–425 nm originated from the π-π* transition of the polymer backbone. The second absorption band at longer wavelength in the range of 425–650 nm was attributed to intramolecular charge transfer (ICT) interactions between the polymer main chains and acceptor end groups of the conjugated side chain [18]. An increase of the strong electron-withdrawing substituent (cyano or benzothiazolyl group) on the conjugated side chain as acceptor end groups led to a red-shift of the ICT absorbance peak. Therefore, PMA-DCN and PMA-CNB showed more red-shifted ICT absorbance peaks than PMA-CNR. The red-shift phenomenon can also be ascribed to the planarity of the acceptor end groups on the conjugated side chain, which increase ICT interactions to the polymer main backbone.

A similar trend was found in PMAT-series copolymers, whereby PMAT-DCN and PMAT-CNB exhibited more red-shifted ICT absorbance peaks than PMAT-CNR. Notably, by extending the conjugated length of the side chain on the triphenylamine unit, PMAT-series copolymers exhibited a significant red-shift and a broader absorption range than PMA-series copolymers, which implies that ICT interactions are enhanced when the conjugated length of the side chain is increased. In thin film state, the absorption spectra of both copolymer series are red-shifted and broader than those in solution state, indicating that the π-π interchain interactions of polymer chains are enhanced in thin film state. Broad absorption is favorable for harvesting solar light and expected to improve the absorption efficiency of the photo-active layer. Moreover, strong π-π interactions facilitate charge transport in devices, thereby increasing the induced photocurrent in PSCs. These results suggest that the photophysical properties of these copolymers can be tuned by manipulating the acceptor end groups in the side chains.

### 3.3. Electrochemical Properties

**Figure 3:**
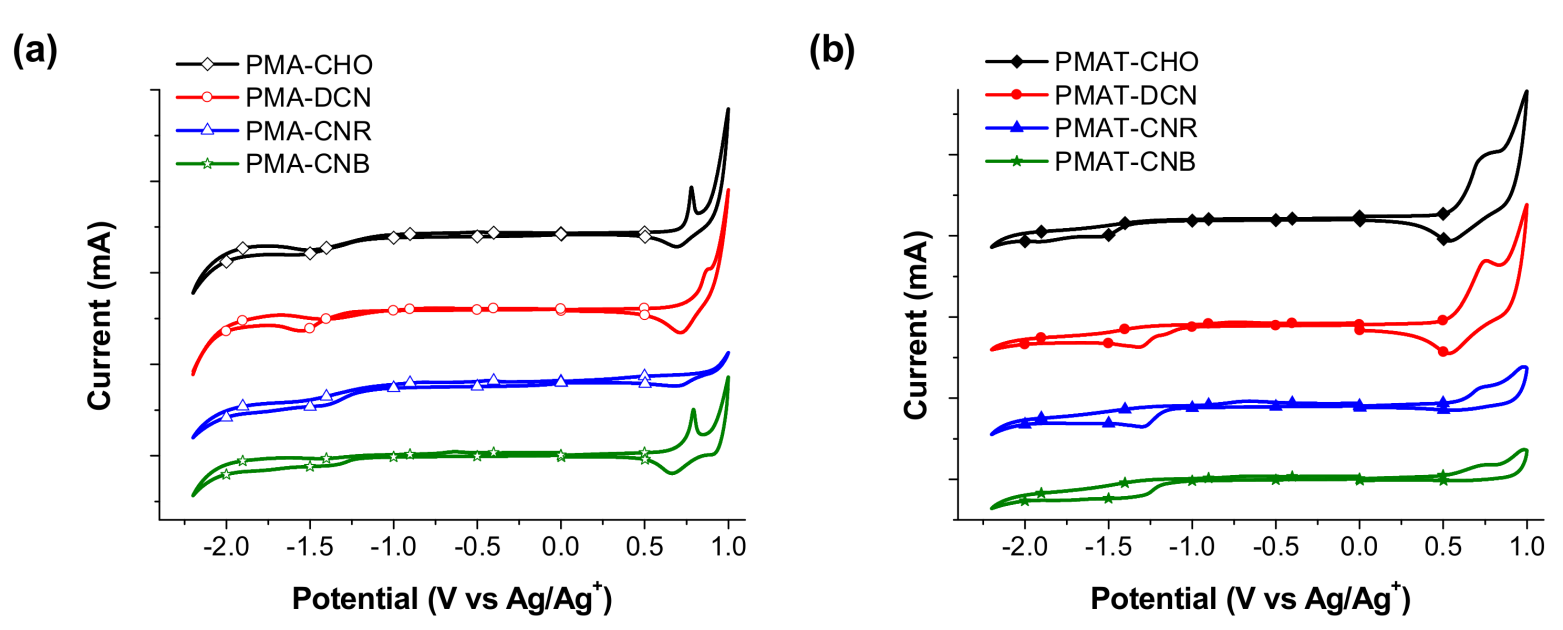
Cyclic voltammograms of copolymer films in Bu_4_NClO_4_ solution (0.1 M in MeCN) for (**a**) PMA-CHO, PMA-DCN, PMA-CNR and PMA-CNB; (**b**) PMAT-CHO, PMAT-DCN, PMAT-CNR and PMAT-CNB.

Cyclic voltammetry (CV) was employed to investigate the HOMO and LUMO energy levels of these copolymers. Figure 3 shows the oxidation and reduction behaviors revealed by CV measurements of the copolymers. The HOMO and LUMO values of these copolymers were estimated from the potentials at the onset of oxidation and reduction, and the results are also summarized in Table 2. The HOMO and LUMO levels of PMA-CHO were ‒5.44 and ‒3.43 eV, respectively. When various acceptor end groups were attached onto the precursor copolymer (PMA-CHO), the HOMO levels of PMA-DCN, PMA-CNR, and PMA-CNB were ‒5.46, ‒5.45, and ‒5.42 eV, respectively, while LUMO levels were ‒3.65, ‒3.70, and ‒3.62 eV, respectively. The HOMO levels of PMA-DCN, PMA-CNR, and PMA-CNB were almost unchanged, whereas their LUMO levels were significantly lowered, indicating that the LUMO levels of these copolymers can be easily tuned by attaching different acceptor end groups onto the precursor copolymer, which is consistent with the results relating to photophysical properties. 

Similarly, the same trend was observed in PMAT-series copolymers. The HOMO and LUMO levels of PMAT-CHO were ‒5.30 and ‒3.44 eV, respectively. After PMAT-CHO was functionalized by various acceptor end groups, the HOMO levels of PMAT-DCN, PMAT-CNR, and PMAT-CNB became ‒5.29, ‒5.30, and ‒5.31 eV, respectively, while LUMO levels were ‒3.80, ‒3.76, and ‒3.78 eV, respectively. The HOMO levels remained nearly the same as compared with PMAT-CHO, but LUMO levels decreased. Notably, by extending the conjugated length of the side chain on the triphenylamine moiety, the decrease in LUMO levels of PMAT-series copolymers is greater than that of PMA-series copolymers. This is attributed to the thiophene ring on the PMAT-series copolymers not only extending the side chain conjugated length but also providing delocalization of π-electrons between triphenylamine and various acceptor ends, lowering the orbital energy. This enhances the electron-withdrawing ability of the acceptors and results in lower LUMO levels than in PMA-series copolymers. This implies that the LUMO levels of the copolymers can be further tuned by extending the conjugated length of side chains on triphenylamine units. The reduced energy band gap is also responsible for increasing the spectral absorption at long-wavelength region.

### 3.4. PV Properties of PSCs

**Figure 4:**
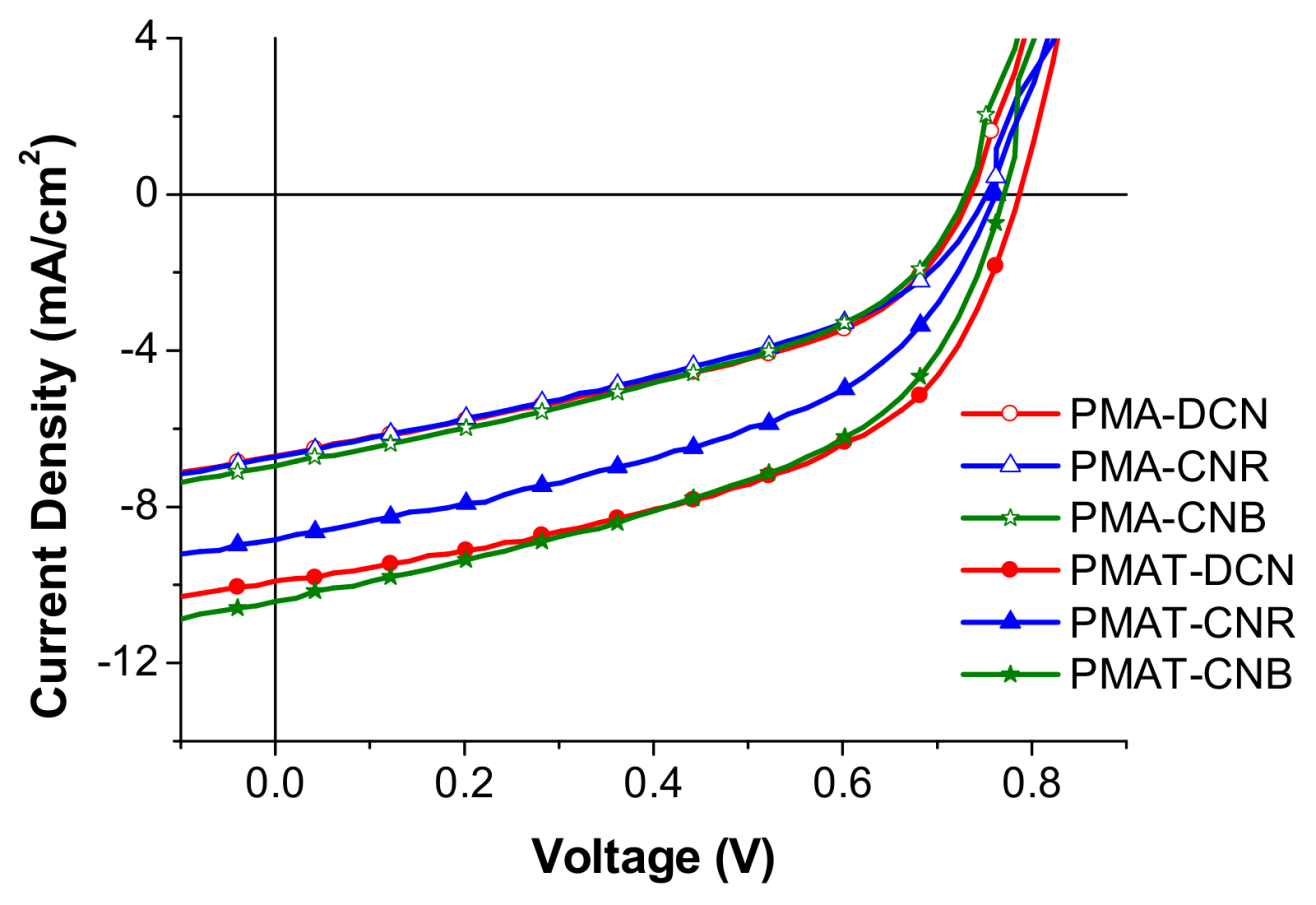
*J-V* characteristics of copolymer/PC_71_BM bulk heterojunction solar cells under illumination of AM 1.5 G, 100 mW cm^‒2^.

BHJ PSCs were fabricated using PMA- and PMAT-series copolymers as the donor and PC_71_BM as the acceptor, with the typical structure of an ITO/PEDOT:PSS/copolymer: PC_71_BM/Ca/Al for examining the photovoltaic properties of these copolymers. The typical photocurrent density-voltage (*J-V*) characteristics of the PSCs measured under simulated AM 1.5 G illumination (100 mW/cm^2^) are plotted in Figure 4, and the relevant average photovoltaic parameters including *V_oc_*, *J_sc_*, fill factor (FF), and PCE, are listed in Table 3. The optimum PCEs for PMA-DCN, PMA-CNR, and PMA-CNB PSCs were 2.14, 2.05, and 2.16%, respectively. For the PMA-series copolymers, the *J_sc_* was in the range of 6.70 to 6.95 mA cm^−2^, the *V_oc_* was from 742 to 766 mV, and FF was from 39.86 to 43.02%. PMA-CNB exhibited the highest *J_sc_*, which is ascribed to its broad absorption region resulting from progressively enhanced ICT interactions. Thus, the *J_sc_* was increased, as shown by the absorption spectra. The *V_oc_* values of these devices based on PMA-series copolymers were nearly the same despite various acceptor groups being attached onto the copolymer. This is ascribed to the fact that HOMO levels of PMA-series copolymers remain almost unchanged, and similar phenomena have been observed in the literature [18,34].

Much higher PCEs were obtained from the PSCs synthesized using PMAT-series copolymers. The PCEs for PMAT-DCN, PMAT-CNR, and PMAT-CNB PSCs were 4.01, 3.14, and 3.82%, respectively, with corresponding *J_sc_* values of 9.89, 8.84, and 10.42 mA cm^−2^, *V_oc_* values of 782, 762, and 769 mV, and FF values of 51.85, 46.55, and 47.73%, respectively. Similarly, the PSC based on PMAT-CNB displayed the highest *J_sc_*, which is ascribed to its relative higher absorption intensity in the absorption spectra. However, although the *V_oc_* values of the PSCs based on PMAT-series copolymers were almost the same, they were higher than those of the devices based on PMA-series copolymers, which is likely due to a non-optimal morphology in PMA-series copolymer blend films [38,39,40].

**Figure 5:**
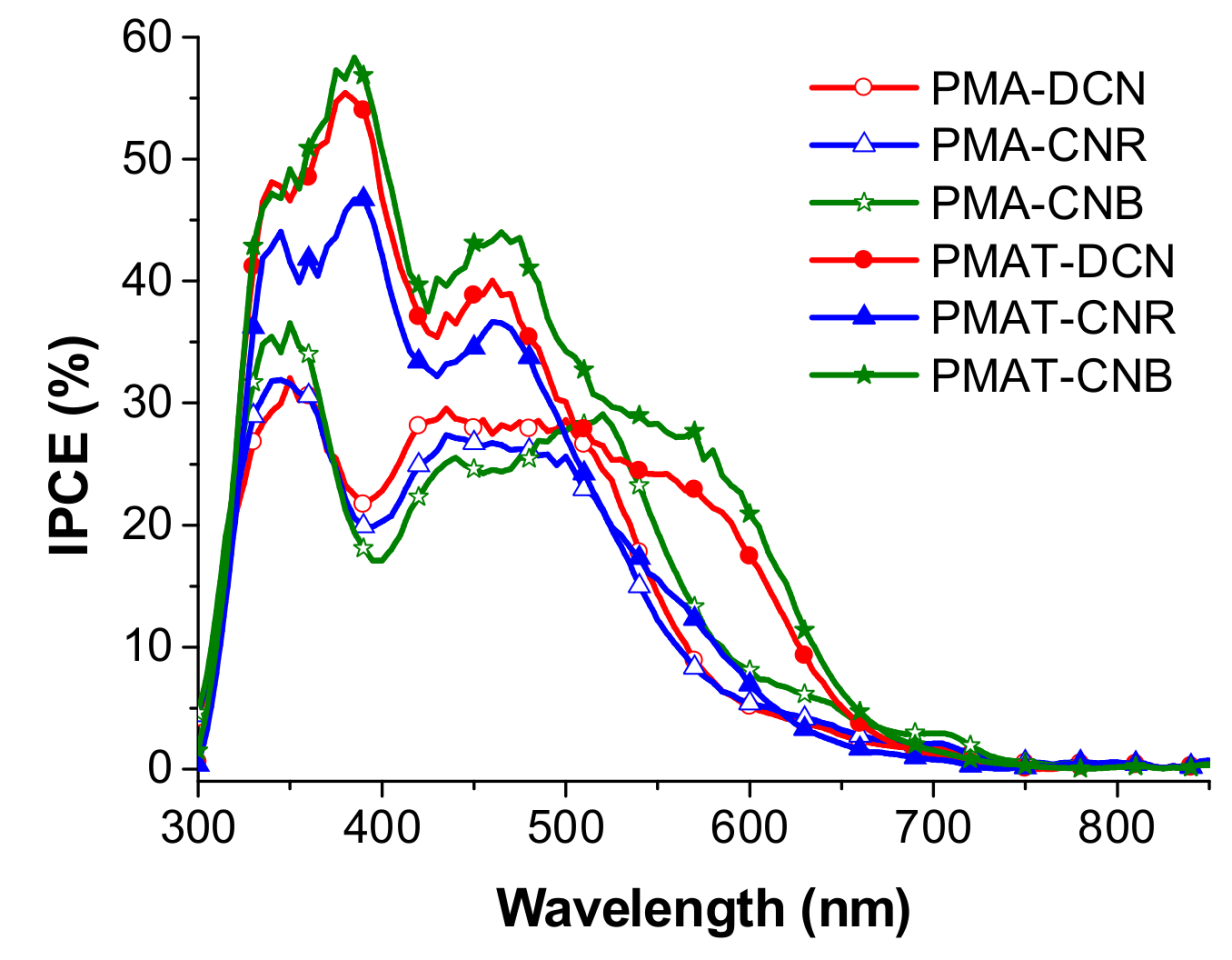
Incident photon-to-electron conversion efficiency (IPCE) spectra of the polymer solar cells (PSCs) with copolymer:PC_71_BM = 1:2 as the active layer.

Interestingly, with the addition of a thiophene ring in the side chains of the triphenylamine unit, the overall device performance was enhanced. This implies that PMAT-series PSCs exhibited higher PCEs than PMA-series PSCs, which is attributed to the wide absorption range of PMAT-series copolymers that increased absorption in the long-wavelength region. In addition, the relatively low LUMO levels of PMAT-series copolymers decrease the energy difference between the copolymer and PC_71_BM, which is beneficial for charge separation at the donor/acceptor interface. These results indicate that subtle tuning of the chemical structure of a copolymer can significantly influence the device performance. To confirm the accuracy of the device measurements, the corresponding incident photon-to-electron conversion efficiency (IPCE) spectra of these copolymer/PC_71_BM blend films were measured and are shown in Figure 5. The IPCE profiles of these devices are consistent with the corresponding absorption spectra of copolymers and agree well with the *J_sc_* values obtained from *J-V* measurements, indicating that the broader absorption region and higher external quantum efficiency value contribute to photocurrent generation and the higher resulting PCE of the device. In addition, the *J_sc_* values that obtained by integration of IPCE curves (Table 3) show similar trend with that from the *J-V* measurements, this implies the photovoltaic results are reliable. The carrier mobility also plays an important role in determining the overall device photovoltaic performance, thus the hole-only devices with device configuration of ITO/PEDOT:PSS/polymer:PC_71_BM/Au were fabricated, and the hole mobility (μ_SCLC,h_) was estimated using the SCLC method (Supporting information, Figure S1 and Table S1). The calculated μ_SCLC,h_ for PMA-DCN/PC_71_BM, PMA-CNR/PC_71_BM, and PMA-CNB/PC_71_BM blend films were 6.94 × 10^−5^, 2.15 × 10^−5^, and 3.58 × 10^−5^, respectively, while the μ_SCLC,h_ for PMAT-DCN/PC_71_BM, PMAT-CNR/PC_71_BM, and PMAT-CNB/PC_71_BM blend films were 5.07 × 10^−4^, 1.90 × 10^−4^, and 3.22 × 10^−4^, respectively. The hole mobilities showed clear dependence on the *J-V* results, indicating high hole mobility of blend film is beneficial for charge transport, and thus the PCEs of PSCs were enhanced. Notably, the PMAT-series copolymers/PC_71_BM blend films displayed slightly higher μ_SCLC,h_ than that of PMA-series copolymers/PC_71_BM blend films, implying that with addition of a thiophene ring in the side chains of the triphenylamine unit not only increase the spectral absorption at long-wavelength region, but also enhance the carrier mobilities of the blend films.

### 3.5. Transmission Electron Microscopy Investigation

**Figure 6:**
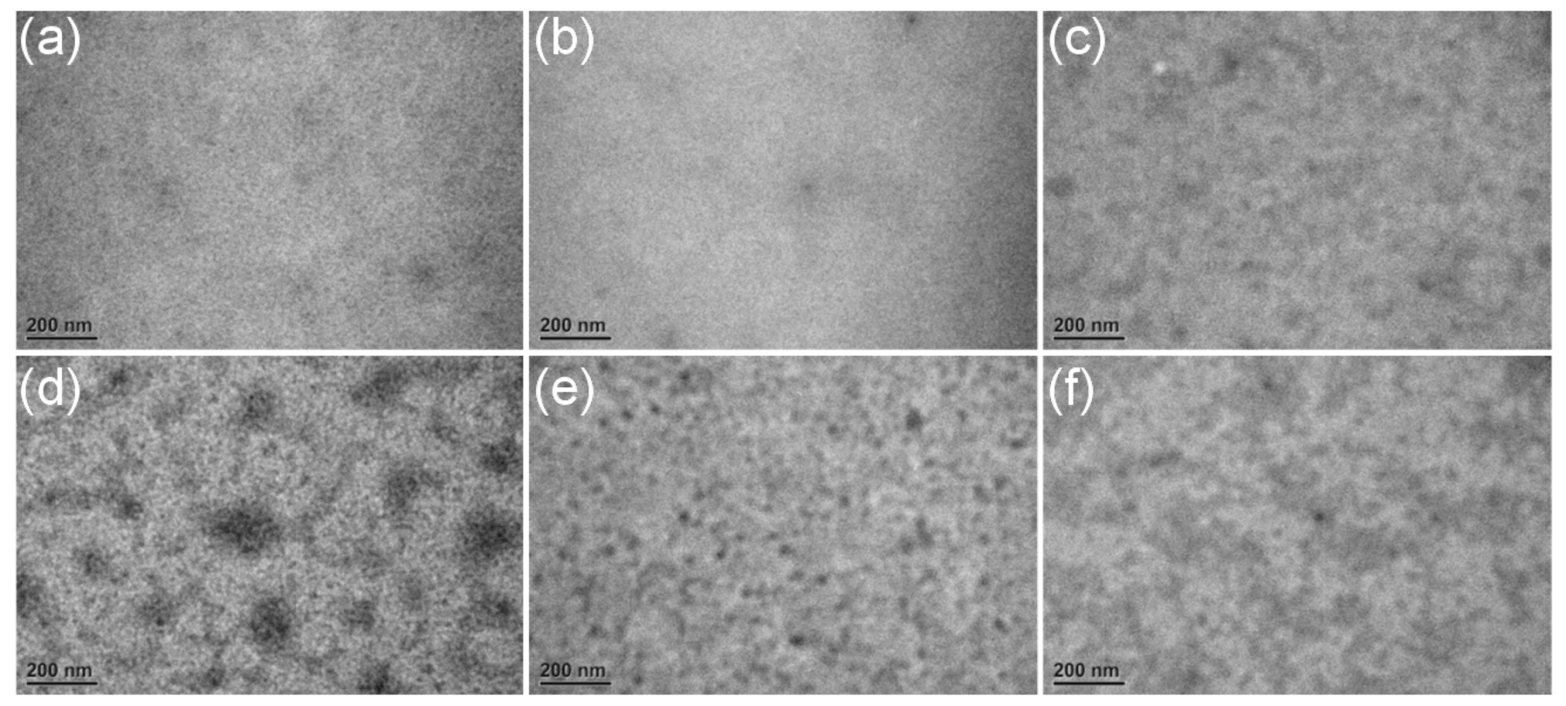
Transmission electron microscopy (TEM) images of polymer:PC_71_BM blend films for (**a**) PMA-DCN; (**b**) PMA-CNR; (**c**) PMA-CNB; (**d**) PMAT-DCN; (**e**) PMAT-CNR; and (**f**) PMAT-CNB.

To determine differences in the photocurrent of the devices, the surface morphology of active layers was analyzed using TEM images to gain further insight into these blend films. Figure 6 shows TEM images of blend films that are identical to the active layers of the devices listed in Table 3. For PMA-series blend films, the PMA-DCN and PMA-CNR blend films exhibited smoother features than those of PMA-CNB, which implies that less phase separation occurs in PMA-DCN and PMA-CNR blend films. Better microphase separation would be beneficial for more effective exciton migration to the donor/acceptor interface for charge separation [37]. As a result, the PSC based on PMA-CNB displayed a higher *J*_sc_. In contrast, the PMAT-DCN and PMAT-CNB blend films exhibited bicontinuous interpenetrating networks, which is beneficial for exciton dissociation and charge transport, leading to high *J*_sc_ and FF and hence high PCE. The PMAT-CNR blend film presented mild phase separation domains with clear agglomerates; therefore, the PSC based on PMAT-CNR showed medium device performance among the PMAT-series copolymers. Both the PMA-CNR and PMAT-CNR blend films exhibited a relatively homogeneous dispersion compared to the others in each series, which was ascribed to the alkyl chain on the acceptor groups, leading to improved solubility. Notably, the PMAT-series blend films exhibited clearer phase separation morphology than PMA-series blend films, indicating that device performance is strongly affected by blend film morphology, which agrees well with the results obtained from *J*-*V* characteristics and IPCE results.

## 4. Conclusions

In summary, we synthesized two series (PMA and PMAT) of two-dimensional donor-acceptor copolymers consisting of a 3,4-bis(4-bromophenyl)maleimide derivative, and triphenylamine with a conjugated side chain with various acceptors attached as end groups, for PSC applications. By varying the electron-withdrawing strength of acceptor end groups on the conjugated side chain of the triphenylamine moiety, the ICT absorption peaks and LUMO levels of these copolymers were significantly affected. In addition, by extending the side chain length on the triphenylamine unit, the absorption spectra of PMAT-series copolymers were more red-shifted and broader, and device performances was enhanced compared to PMA-series copolymers. BHJ PSCs composed of PMA-series copolymers and PC_71_BM with a weight ratio of 1:2 had PCEs ranging from 2.05 to 2.16%, while PSCs based on PMAT-series copolymers exhibited PCEs ranging from 3.14 to 4.01%. These enhancements in the PCE of PSCs based on PMAT-series copolymers originate mainly from *J*_sc_ values, which can be ascribed to light absorption enhancement, as confirmed by the UV-vis spectra, TEM images, and IPCE results. The relatively low LUMO levels of PMAT-series copolymers, which decrease the energy difference between the copolymer and PC_71_BM that benefits charge separation at the donor/acceptor interface, may also enhance *J*_sc_ values. These results provide a strategy for manipulating the photophysical and electric properties of copolymers, simply by attaching various acceptor end groups on the side chains. This research also indicates that subtle tuning of the chemical structure can significantly influence device performance. With success in direct C–H arylation reaction using 3,4-bis(4-bromophenyl)maleimide derivative, further design constructing by this monomer with fully conjugated D-A structure and improved efficiency will be investigated in the near future.

## Figures and Tables

**Table 1:** Molecular weights and thermal properties of copolymers.

Copolymers	*M*_n_^a^ (g/mol)	*M*_w_^a^ (g/mol)	PDI (*M*_w_/*M*_n_)	*T*_d_^b^ (°C)
PMA-CHO	7620	9220	1.21	418
PMA-DCN	8830	11,480	1.30	385
PMA-CNR	7830	11,350	1.45	325
PMA-CNB	7390	10,200	1.38	419
PMAT-CHO	7880	9300	1.18	388
PMAT-DCN	8200	11,890	1.45	421
PMAT-CNR	9600	13,350	1.39	346
PMAT-CNB	8020	12,110	1.51	410

^a^ *M*_n_, *M*_w_ and polydispersity index (PDI) of the polymers were determined by GPC using polystyrene standards in THF; ^b^ Temperature of 5% weight loss.

**Table 2:** Photophysical and electrochemical properties of copolymers.

Copolymers	In Solution ^a^ (nm)	In Film ^b^ (nm)	HOMO/LUMO ^c^ (eV)	$E_{g}^{ec}$ ^d^ (V)
	$\lambda_{max}^{abs}$	$\lambda_{max}^{abs}$		
**PMA-CHO**	374	372	−5.44/−3.43	2.01
**PMA-DCN**	355, 455	358, 465	−5.46/−3.65	1.81
**PMA-CNR**	363, 448	363, 452	−5.45/−3.70	1.75
**PMA-CNB**	360, 470	365, 483	−5.42/−3.62	1.80
**PMAT-CHO**	358, 442	362, 444	−5.30/−3.45	1.86
**PMAT-DCN**	363, 501	367, 513	−5.29/−3.80	1.49
**PMAT-CNR**	367, 472	371, 486	−5.30/−3.76	1.54
**PMAT-CNB**	367, 484	371, 493	−5.31/−3.78	1.53

^a^ Measured in toluene solution; ^b^ Casted from dichloromethane solution.; ^c^ The energy levels were calculated according to: Highest occupied molecular orbital (HOMO) = −e($E_{on}^{ox}$ − $E_{on}^{ferrocene}$ + 4.8) (eV), lowest unoccupied molecular orbital (LUMO) = −e($E_{on}^{re}$ − $E_{on}^{ferrocene}$ + 4.8) (eV); ^d^ Estimated using empirical equations: $E_{g}^{ec}$ = $E_{HOMO}$ − $E_{LUMO}$.

**Table 3:** Photovoltaic performances of devices under the illumination of AM 1.5 G, 100 mW cm^−^^2^.

Active Layer	*V_oc_* (mV)	*J_sc_* [*J_sc_*] ^a^ (mA/cm^2^)	FF (%)	PCE (%)	Thickness (nm)
**PMA-DCN**	742	6.70 [6.41]	43.02	2.14	95
**PMA-CNR**	766	6.72 [6.59]	39.86	2.05	110
**PMA-CNB**	742	6.95 [7.09]	41.93	2.16	102
**PMAT-DCN**	782	9.89 [9.85]	51.85	4.01	113
**PMAT-CNR**	762	8.84 [8.72]	46.55	3.14	90
**PMAT-CNB**	769	10.42 [10.36]	47.73	3.82	121

^a^ Values calculated from IPCE spectra.

## Supplementary Materials

The following are available online at http://www.mdpi.com/2073-4360/10/4/384/s1. Figure S1: Space-charge-limited *J*-*V* curves of hole-only devices based on copolymer/PC_71_BM blend films; Table S1: Calculated hole mobilities of copolymer/PC_71_BM blend films.
