# Supplementary material for: Structure-Function Relationships in PMA and PMAT Series Copolymers for Polymer Solar Cells
Source: Polymers (Basel). 2018 Apr 1;10(4):384. doi: 10.3390/polym10040384 (PMC6415455; doi:10.3390/polym10040384)
Supplement: Supplementary file 1 [file polymers-10-00384-s002.pdf]

Electronic Supplementary Information (ESI) for:

Structure-Function Relationships in PMA and PMAT Series Copolymers for Polymer Solar Cells

Jhe-Han Chen, Chi-Kan Liu, Wei-Che Chang, Pai-Tao Sah, Li-Hsin Chan*

Department of Applied Materials and Optoelectronic Engineering, National Chi Nan University, Nantou, Taiwan 54561, ROC.

*To whom all correspondence should be addressed.

Chan* is to be contacted at the Department of Applied Materials and Optoelectronic Engineering, National Chi Nan University, Nantou Taiwan 54561, ROC. Tel.: +886-49-2910960-4908.

E-mail addresses: lhchan@ncnu.edu.tw (L.-H. Chan)

Supplementary Information

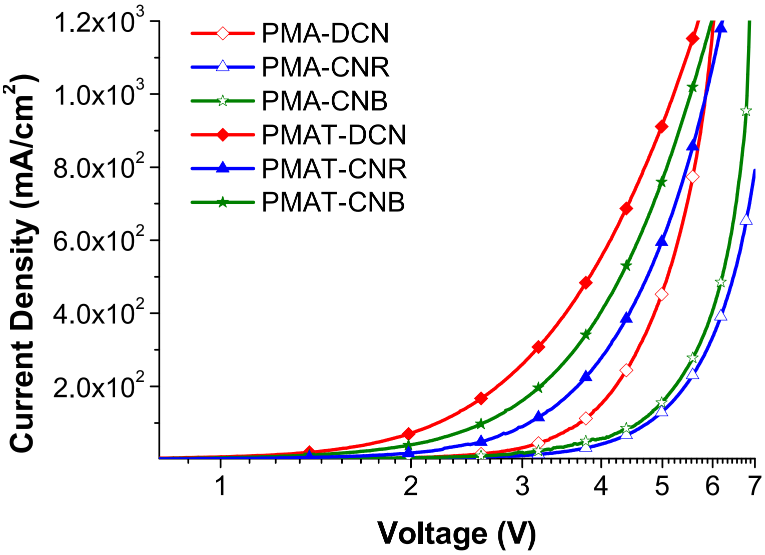

Figure S1. Space-charge-limited *J-V* curves of hole-only devices based on copolymer/$PC_{71}BM$ blend films.

Table S1. Calculated hole mobilities of copolymer/$PC_{71}BM$ blend films.

Copolymer/$PC_{71}BM$ (w/w = 1:2)	$\mu_{SCLC,h}$ ($cm^2V^{-1}S^{-1}$)
PMA-DCN	6.94×10^{-5}
PMA-CNR	2.15×10^{-5}
PMA-CNB	3.58×10^{-5}
PMAT-DCN	5.07×10^{-4}
PMAT-CNR	1.90×10^{-4}
PMAT-CNB	3.22×10^{-4}
